# An Approach for Systems-Level Understanding of Prostate Cancer from High-Throughput Data Integration to Pathway Modeling and Simulation

**DOI:** 10.3390/cells11244121

**Published:** 2022-12-19

**Authors:** Mohammad Mobashir, S. Pauliina Turunen, Mohammad Asrar Izhari, Ibraheem Mohammed Ashankyty, Thomas Helleday, Kaisa Lehti

**Affiliations:** 1 Department of Microbiology, Tumor and Cell Biology, Karolinska Institutet, Solnavägen 9, Solna 17165, Sweden; 2 Faculty of Applied Medical Sciences, University of Al-Baha, Al-Baha 65528, Saudi Arabia; 3 Department of Medical Laboratory Technology, Faculty of Applied Medical Sciences, King Abdulaziz University, Jeddah 22233, Saudi Arabia; 4 SciLifeLab, Department of Oncology and Pathology, Karolinska Institutet, P.O. Box 1031, 17121 Stockholm, Sweden

**Keywords:** systems-level understanding, high-throughput data, crosstalk, mathematical modeling and simulation, docking, gene expression, enriched pathways

## Abstract

To understand complex diseases, high-throughput data are generated at large and multiple levels. However, extracting meaningful information from large datasets for comprehensive understanding of cell phenotypes and disease pathophysiology remains a major challenge. Despite tremendous advances in understanding molecular mechanisms of cancer and its progression, current knowledge appears discrete and fragmented. In order to render this wealth of data more integrated and thus informative, we have developed a GECIP toolbox to investigate the crosstalk and the responsible genes’/proteins’ connectivity of enriched pathways from gene expression data. To implement this toolbox, we used mainly gene expression datasets of prostate cancer, and the three datasets were GSE17951, GSE8218, and GSE1431. The raw samples were processed for normalization, prediction of differentially expressed genes, and the prediction of enriched pathways for the differentially expressed genes. The enriched pathways have been processed for crosstalk degree calculations for which number connections per gene, the frequency of genes in the pathways, sharing frequency, and the connectivity have been used. For network prediction, protein–protein interaction network database FunCoup2.0 was used, and cytoscape software was used for the network visualization. In our results, we found that there were enriched pathways 27, 45, and 22 for GSE17951, GSE8218, and GSE1431, respectively, and 11 pathways in common between all of them. From the crosstalk results, we observe that focal adhesion and PI3K pathways, both experimentally proven central for cellular output upon perturbation of numerous individual/distinct signaling pathways, displayed highest crosstalk degree. Moreover, we also observe that there were more critical pathways which appear to be highly significant, and these pathways are HIF1a, hippo, AMPK, and Ras. In terms of the pathways’ components, GSK3B, YWHAE, HIF1A, ATP1A3, and PRKCA are shared between the aforementioned pathways and have higher connectivity with the pathways and the other pathway components. Finally, we conclude that the focal adhesion and PI3K pathways are the most critical pathways, and since for many other pathways, high-rank enrichment did not translate to high crosstalk degree, the global impact of one pathway on others appears distinct from enrichment.

## 1. Introduction

High-throughput data ((epi-)genomic, transcription, and proteomic) are frequently generated with the goal to understand the genotype–phenotype relationship in complex diseases [1,2,3]. The initial goal in the analysis of these high-throughput data remains the identification of a list of important targets (genes/proteins). Once the target genes/proteins are identified then the major challenge remains at functional annotation and pathway-level understanding [1,2,3,4,5,6]. In this direction, multiple successful approaches have been developed. Among the most frequently used tools for functional annotation for a given gene list are DAVID [7], EnrichNet [8], and PANTHER [9]. So far, in most high-throughput studies, the major focus has been on functional classification and annotation of genes or the analysis of enriched pathways. In cancer, mutations and copy number alterations coupled with epigenetic aberrations and altered gene expression are commonly focused points of study towards understanding the cause, heterogeneity, and drug resistance or defining other phenomena related to the aggressiveness and progression of cancers [10,11]. Accordingly, compared to normal cells, drastic changes occur in cancer cells at multiple levels, including molecular functions and cellular processes [12,13,14,15,16,17,18,19,20,21,22,23,24]. In different cancer types, the pattern of aberrations behind the disrupted tissue and cell functions varies substantially [25,26]. High-throughput identification of the different gene expression patterns and genomic changes are frequently used to uncover the biological pathways involved. From there, it is vital to understand which are the functionally most significant individual changes and pathway-level alterations [1,2,3,27]. To achieve such goals, DAVID, EnrichNet, and PANTHER are helpful. As a result, incorporating multi-omic data in a meaningful way to provide a more comprehensive analysis of a biological area of interest is becoming more common [28,29,30,31,32,33,34,35]. A critical and promising challenge which remains unanswered is how the significantly affected or enriched pathways in a particular disease type or even for the same disease in an individual patient collectively coordinate or crosstalk with each other [31,32,33,36,37,38,39,40].

To address this, we have here developed a GECIP toolbox, a computational method that first processes the list of genes or proteins of interest and performs pathway enrichment and network analysis similar to other methods [30]. Most importantly, and unlike the previously developed approaches [4,5,6,41,42,43,44,45,46,47,48], GECIP calculates the predictive degree of crosstalk ($CT_{degree}$) between the pathways as the final output. This toolbox is different from the existing previous similar work in the sense that it includes the list of potential genes or targets and proceeds for pathway enrichment analysis, network-level understanding of the genes or the proteins and enriched pathways, and finally, associates the different parameters (list of genes/proteins, connectivity, sharing frequency of genes/pathways with genes/proteins/pathways) to calculate the possible interactions between the enriched pathways.

**Figure 1:**
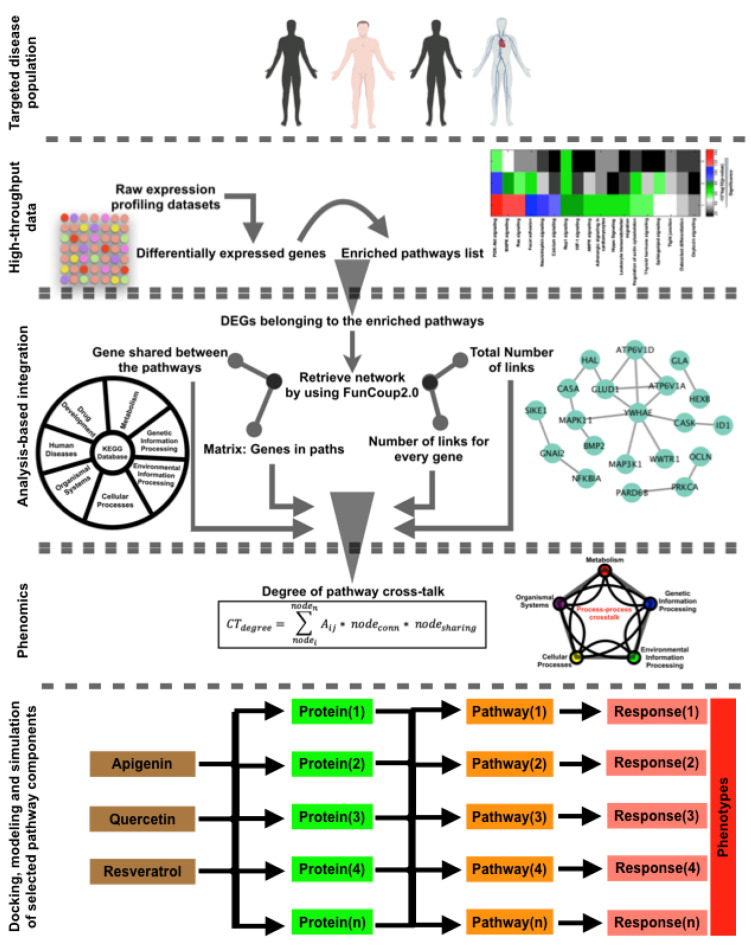
GECIP (Gene set, Enrichment, and Crosstalk between the Inferred Pathways) workflow for pathway enrichment analysis and crosstalk degree calculation for the enriched pathways.

In this work, the GECIP toolbox was applied for the differentially expressed genes (DEGs) in prostate cancer versus normal tissue. Since the gene expression alterations in different cancer patients and experimental conditions may vary, we have collected three separate human microarray gene expression datasets for prostate cancer (gene expression omnibus; https://www.ncbi.nlm.nih.gov/geo/ (accessed on 1 November 2022)). The steps in GECIP proceed from preparation of a gene list (DEGs), formulation of the network of DEGs, prediction and analysis of enriched KEGG pathways [1,2,3,7,49], and finally, calculation of the $CT_{degree}$ between the pathways (Figure 1).

## 2. Materials and Methods

**Data collection and processing:** In this work, we have collected prostate cancer gene expression datasets of normal and cancerous tissue from Gene Expression Omnibus (GEO) [50]. Three sets of datasets for prostate cancer studies have been collected. These gene expression profiling datasets were Affymetrix Human Genome U133 Plus 2.0 Array, Affymetrix Human Genome U133A, and Affymetrix Human Genome U133B, respectively. For generating the DEG network, FunCoup2.0 [51] has been used for all the networks throughout the work, and cytoscape [52] has been used for network visualization [53]. For most of our coding and calculations, MATLAB has been used. FunCoup predicts four different classes of functional coupling or associations such as protein complexes, protein–protein physical interactions, metabolic, and signaling pathways [1,2,3,4,5,6,51].

**Analysis of crosstalk between the pathways:** To investigate the crosstalk between the enriched pathways, we have divided the approach into three major steps. In the first step we have processed the raw data for normalization applying RMA [7,54]. A standard statistical t-test (*mattest* MATLAB function) has been applied for identifying significant changes between two groups of data (normal or control and cancer or target) for differential gene expression analysis [8,15,16,17,18,19,22,23,24,53,55,56,57]. To assess the differences in gene expression between two experimental conditions or phenotypes, *mattest* (https://www.mathworks.com/help/bioinfo/ref/mattest.html, (accessed on 1 November 2022)) uses a two-sample t-test. Every gene is subjected to a standard two-tailed, two-sample unpaired t-test for differential expression, which results in a *p*-value for every gene. For FDR calculation, the details could be seen in the link mentioned here (https://se.mathworks.com/help/bioinfo/ref/mafdr.html, (accessed on 1 November 2022)). For fold change calculation, we have used the *p*-values after corrections, and the threshold was 0.05. The same we have also updated in the main text. In the second step, we have prepared the list of DEGs and the total gene list and have also collected the list of KEGG pathways. Once we have these three lists in normal text file format, a Fischer exact test [9,58,59] is performed as follows:

(1) $$f\left(k;N,\;m,\;n\right)=\frac{\left(\begin{array}{c} m\\ k \end{array}\right)\left(\frac{N-m}{n-k}\right)}{\left(\frac{N}{n}\right)}$$

where *m* is the total number of genes genome-widely annotated with each pathway, *N* is the total number of genome-wide genes, *k* is the number of genes annotated with an individual pathway in the gene list, *n* is the total number of genes in the gene list, and *f-value* is the value of probability that this random event could happen under hypergeometric distribution [10,11,55,60,61]. To get the probability of more extreme cases, we sum up all the probability as follows:

(2) $$p=\overset{n}{\underset{k}{\sum }}f\left(k;N,\;m,\;n\right)$$

For all the pathways listed in the KEGG database (from five classes, as shown in Figure 1), we continue this step. After calculating *p*-values, multiple hypothesis testing has been applied for false discovery rate (FDR) correction. From this step, we get the list of enriched pathways. We have considered only those pathways which have FDR < 0.05 [12,62].

Finally, in the third step, after calculating the enriched KEGG pathways, we proceed to calculate the crosstalk between the enriched pathways. For crosstalk analysis between all the enriched pathways, an adjacency matrix ($A_{ij}$) is generated for the network, shown in Figure 3c. This $A_{ij}$ stands for the connectivity, which means the specific node connecting or not [63,64,65,66,67,68]. Afterward, the degree of crosstalk is calculated by using a mathematical equation (Equation (3)).

(3) $$CT_{degree}=\overset{node_{n}}{\underset{node_{i}}{\sum }}A_{ij}*\;node_{conn}*\;node_{sharing}*\;\frac{Conn_{total}}{\left(node_{s}\left(conn\right)+node_{t}\left(conn\right)\right)}$$

Here, $CT_{degree}$ is crosstalk degree between two pathways, $node_{conn}$ is the number of nodes associated with the selected node, and $node_{sharing}$ means how many pathways share the selected nodes. $Conn_{total}$ represents total connectivity within the DEGs’ network, $node_{s}\left(conn\right)$ stands for total number of links for source node, and $node_{t}\left(conn\right)$ stands for total number of links for target node. Thickness of the connecting links between the pathways has been generated based on the $CT_{degree}$, which is based on the overall $CT_{degree}$. The generalized workflow of this work has been presented in Figure 1 (Analysis of crosstalk between the pathways).

**Mathematical modeling and simulation of selected pathway components**: After crosstalk calculations, we have selected a few components which are well-known to play roles in prostate cancer (Figure 6), prepared a mathematical model by using mass-action kinetics and ordinary differential equations (ODEs) [47,69,70,71,72,73,74], and by using an evolutionary algorithm (EA) [68,75], optimized the parameters. The modeling approach and EA optimization approach were adapted from our previous studies [66,67,68]. The summary of the previous approach could be presented here. The interaction matrix between all these signaling molecules, including complexes, is represented as +1 (production/generation), −1 (degradation/dissociation), and 0 (no interaction). The entries of Aij are chosen once for a network under the constraints that the total amount of each protein is conserved and the SN generated does have a stable inactive state. The fitness of a signaling network was tested by calculating its response to six different signals. For every signal, the dynamics of the pre-selected output node is tested whether it exceeds a threshold level of 1/10, defined to be the relative fraction of the dual phosphorylated protein to the total concentration of the protein. If the output node crosses the threshold level at any point during the dynamics, the network gains a fitness contribution. We have applied an evolutionary algorithm to evolve the networks. Before starting evolution, we create a set of diverse networks (N = 200) with the same randomly generated interaction matrix for three proteins with three states (un-, mono-, and dual-phosphorylated) and different randomly selected kinetic parameters. More details of the methodology related to mathematical modeling and evolution of the signaling networks could be seen in our previous studies [66,67,68].

We have established a signaling cascade that operates similarly to the MAPK signaling cascade. The target level is represented by target proteins, which are those proteins that transmit information to the nucleus in the form of the output response. This signaling cascade is divided into several different levels of signaling, such as the receptor level, intracellular signaling level, and the target level. Then, at various signaling levels, we have incorporated various loop types as well as cross-interactions (crosstalk) between two signaling cascades. In the next phase, we have a mass-action kinetic model which utilizes ODEs for all the molecules. The model equation (Equation (4)) used to express the temporal change in the concentration of the signaling molecules was mentioned in detail in our previous work [62,63,64].

(4) $$\frac{dx_{i}}{dt}=\sum^{\;}Production_{rate}-\sum^{\;}Consumption_{rate}$$

We estimated the fitness of all the cascades after calculating the kinetics of all the molecules. Six distinct input signals ($n_{s}=6$) were used for all calculations; 0.0001, 0.001, 0.01, 0.1, 1, and 10 were the six different input signals (different in strength), and for each input signal (n), the kinetics of individual TP is tested as to whether it exceeds the threshold level (threshold level, $TL=\frac{1}{10}th$ of the initial concentration of TP), defined to be the relative fraction of double-phosphorylated form of TP. If this double-phosphorylated form of TP surpasses the threshold level at any time point in a cascade, with or without FBL and FFL, the cascade is given a value of 1.0, known as fitness factor 1 ($F_{factor1}$), else $F_{factor2}=0$. In case of crosstalk of two cascades, in a similar way we check the kinetics of the double-phosphorylated form of TP of both the cascades; if the kinetics of cascade 1 crosses the threshold at any time point, then we assign fitness factor ($F_{factor1}$) a value of 0.5, and if the kinetics of cascade 2 crosses the threshold at any time point, then we assign fitness factor ($F_{factor2}$) a value of 0.5 for cascade 2. After evaluating the kinetics for each cascade for all six input signals (different in strength), we calculate the fitness (F) (as shown in Equation (5)) by taking the mean of the fitness factors ($F_{factor1}\;and\;F_{factor2}$), which can be represented as [66,67,68]:

(5) $$F=\frac{\sum_{n=1}^{n_{s}}F_{factor1}\left(n\right)+\sum_{n=1}^{n_{s}}F_{factor2}\left(n\right)}{n_{s}}$$

We have created a set of cascades (total number of cascades 200) with randomly generated kinetic parameters ($k_{par}$) between 0.001 and 0.1. EA [68,76] has been applied to evolve the signaling cascades. For each cascade, we have F. After calculating F of all the cascades, we select the best 50 cascades (successful cascades) based on higher F values. In order to improve the response kinetics, these successful cascades are allowed to adapt new $k_{par}$. Four copies of all these 50 cascades with updated $k_{par}$ are created to keep the total number of cascades equal in each iteration. All these processes are repeated for 200 iterations. Each iteration is called a generation [66,67,68].

**Docking profiling:** SwissDock has been applied for docking purpose, and the input files were prepared as per SwissDock guidelines [23,77,78]. The ligand structures were collected from PubChem and converted to mol2 format, and the 3D protein structures of the proteins for the selected genes have been predicted by using SWISS-MODEL. After preparing the input files, SwissDock was executed for the default parameters [22,56,57,79,80,81,82].

Experimental validation: We have collected human prostate biopsies for both normal and cancer condition and performed immunohistochemistry (IHC) for human prostate biopsies (normal and cancer). The primary antibodies were p44/42 MAPK (Erk1/2) (L34F12) Mouse mAb #4696 and Phospho-p44/42 MAPK (Erk1/2) (Thr202/Tyr204) (D13.14.4E) XP^®^ Rabbit mAb #4370, both in a ratio of 1:500 (bought from cell signalling). The secondary antibodies were goat anti-mouse immunoglobulin G (IgG)–Alexa Fluor 488 (1:500; Molecular Probes) and goat anti-rabbit IgG–Alexa Fluor 555 (1:500; Molecular Probes).

Details of the staining procedure: These sections were deparaffinized and rehydrated before antigen retrieval with R-Buffer A (Electron Microscopy Sciences) in a pressure cooker. After blocking with 2% bovine serum albumin, the sections were incubated with different primary antibodies (see above) at 4 °C overnight. Extensive rinsing was performed once; the sections were incubated with the secondary antibodies goat anti-mouse immunoglobulin G (IgG)–Alexa Fluor 488 (1:500; Molecular Probes) and goat anti-rabbit IgG–Alexa Fluor 555 (1:500; Molecular Probes) for 1 h at room temperature. DNA was counterstained with TO-PRO-3 iodide (Molecular Probes), and slides were mounted with ProLong Gold (Molecular Probes).

Fluorescence images were obtained with a Zeiss LSM 780 inverted confocal microscope, using a Plan-Apochromat 40×/NA 1.2 objective. Through-focus maximum projection images were acquired from optical sections 0.5 μm apart and with a section thickness of 1.0 μm.

**Figure 2:**
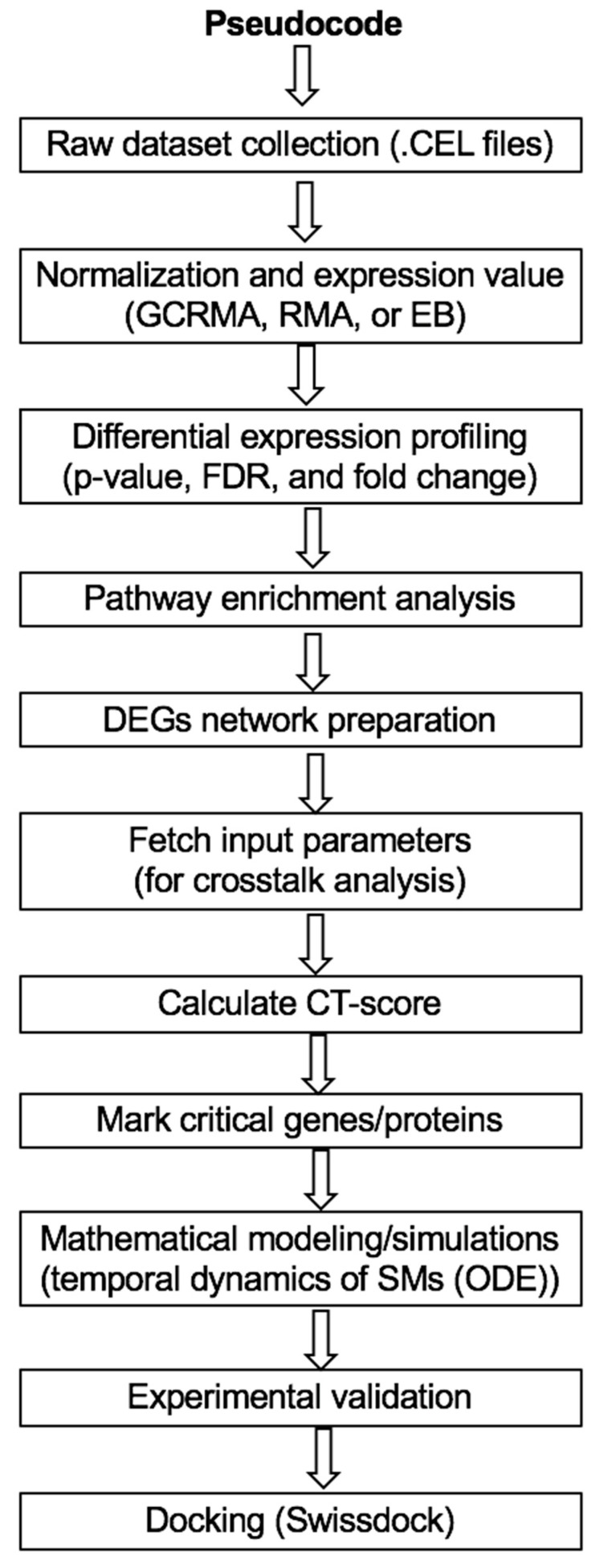
Pseudocode for carry out the entire work presented in this study.

All the MATLAB codes which have been used for entire calculation are available on request, and the pseudocode is presented below (Figure 2).

## 3. Results

### 3.1. Gene Expression Profiling, Enriched Pathways, and Crosstalk Score Calculations

**Figure 3:**
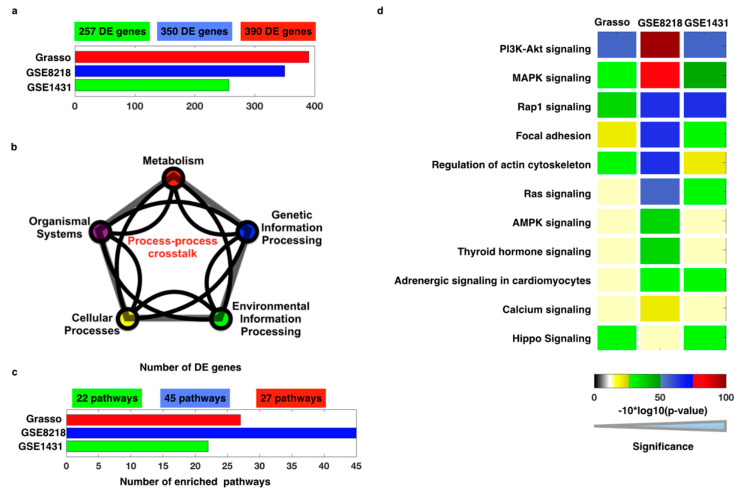
GECIP pathway analysis: (**a**) total number of DEGs for three different datasets of prostate cancer, (**b**) pathways selected from different KEGG pathway classes, (**c**) total number of enriched KEGG pathways for three different datasets of prostate cancer, (**d**) *p*-values (the heat map has been plotted by converting *p*-values into −10 ∗ log10 of *p*-values (red: lowest *p*-value and gray: higher *p*-values and less than 0.05)) of 11 common enriched pathways.

In the first step, the raw microarray gene expression data were processed in order to identify the DEGs. We processed all the raw data through normalization with the robust multi-array average (RMA) method. After normalization, a two-sample *t*-test statistical analysis approach was applied to identify the differentially expressed genes (DEGs) in the three datasets [25,26,55]. In our analysis, we have analyzed the DEGs for all the selected datasets (Figure 3a). With the DEG lists, we next calculated the enriched KEGG pathways and ranked them based on *p*-values (*p* < 0.05; Supplementary Table S1). For pathway enrichment analysis, we have included metabolism, genetic information processing, environmental information processing, cellular processes, and organismal systems, but excluded human diseases and drug development from the altogether seven KEGG database pathway sections (Figure 3b). This exclusion was done to avoid duplication, since the human disease and drug development pathway components are also represented in the five included pathway sections. To calculate $CT_{degree}$, network of DEGs, genes shared between the pathways, total number of links within the DEG network, and number of links for every gene were included as input for the formulated mathematical equation (Figure 1). To acquire these parameters, we used the FunCoup2.0 network (homo sapiens) database [1,2,3,12,27,51]. There, the networks of functional coupling between genes are reconstructed by integrating heterogeneous data (such as mRNA co-expression, phylogenetic profile similarity, protein–protein interaction, protein co-expression, shared transcription factor binding, and domain association data) [10,11,51]. For all the DEGs, we generated the network by FunCoup2.0, and in order to calculate the $CT_{degree}$, the required parameters were extracted from this network and the KEGG database.

We found 27, 45, and 22 enriched pathways in Grasso, GSE8218, and GSE1431, respectively (Figure 3c). Grasso includes a gene expression dataset of prostate cancer samples (Agilent Human Genome 44K), GSE8218 includes gene expression prostate cancer samples (Affymetrix U133A array), and GSE1431 includes gene expression prostate cancer samples (Affymetrix U95Av2). Eleven pathways were common among these three datasets, and most of these pathways are known to be altered with different cancers, including prostate cancer (Figure 3d). Of note, most of the critical pathways with a known association to cancer [4,5,6,8,12,41,42,43,44] were represented in the highly enriched pathway list in different types of cancers [9,83,84]. Among the highly enriched signaling pathways were PI3K-Akt, focal adhesion, regulation of actin cytoskeleton, Ras, Hippo, MAPK, and Rap1 signaling pathways. Furthermore, we have also compared the enriched pathways with four more pathway databases (NCI, Reactome, Wiki, BioCarta), where the majority of our pathways were common (Supplementary Figure S1).

**Figure 4:**
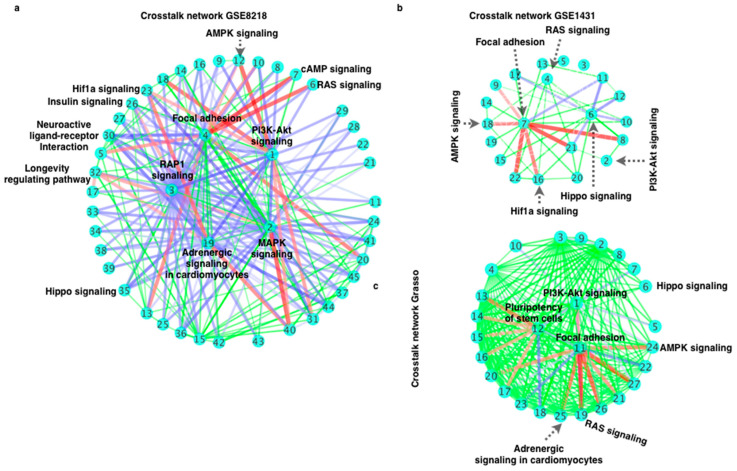
$CT_{degree}$ analysis for the enriched pathways: Degree of crosstalk between the pathways for (**a**) GSE8218, (**b**) GSE1431, and (**c**) Grasso dataset. Different thickness of the edges represents the degree of crosstalk between the pathways (higher the higher the $CT_{degree}$, and lower thickness means lower $CT_{degree}$). Different edges colors are for clarity only.

Since a large number of enriched pathways were evident in all three individual datasets, to unravel more comprehensively the crosstalk between the enriched pathways, all the DEGs of the three datasets were combined into one list, and we further calculated the $CT_{degree}$ for all the enriched pathways (Figure 4a–c).

### 3.2. Pathway–Pathway Interactions (Crosstalk) Analysis for Prostate Cancer Gene Expression Data

**Figure 5:**
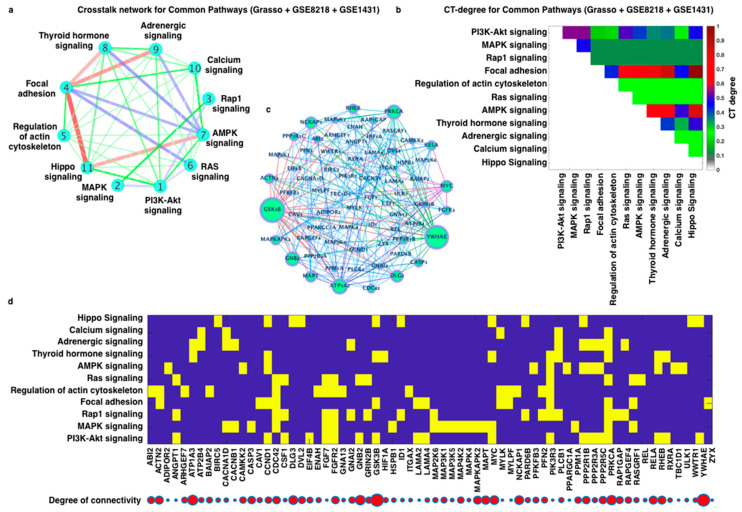
Crosstalk between the common enriched pathways: (**a**) genes belonging (in yellow color) to the crosstalk (pathway–pathway interaction) network and total connectivities for each gene (bigger circle means higher connectivities), (**b**) heatmap to represent the corresponding crosstalk degree between the pathways, (**c**) pathway–pathway interaction network for common enriched pathways among all the three datasets and the pathway components (gene–gene associations), and (**d**) genes shared by the 11 pathways present in pathway–pathway interaction network and their degree of association within the crosstalk network for the corresponding genes.

**Figure 6:**
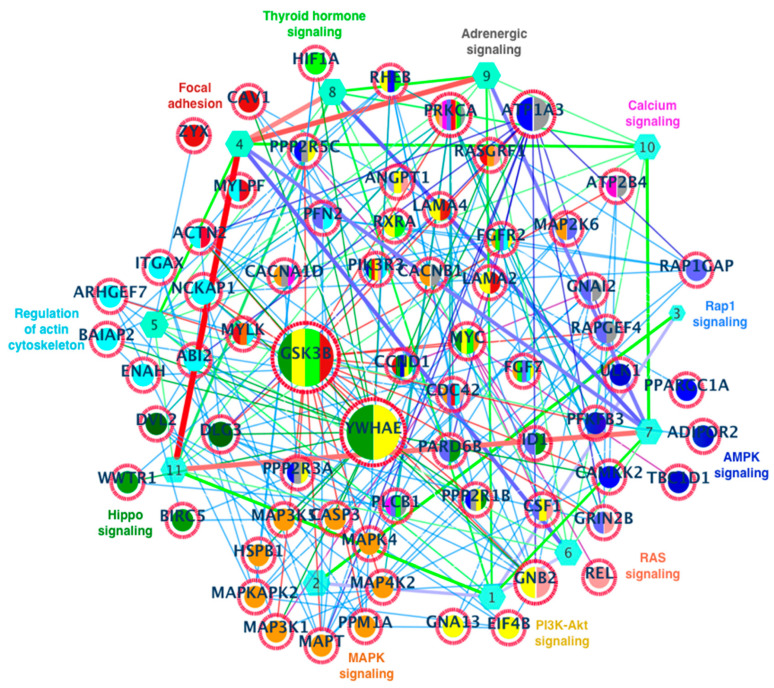
Crosstalk between the common enriched pathways: Pathway–pathway interactions together with the corresponding genes. The hexagonal nodes with number 1–11 represent the pathways, and the genes with more than one color mean that they are shared by more than one pathway. The higher the size of circular nodes (genes), the higher the degree of connectivity.

Many of the enriched pathways were shared between the three datasets, and the pathways with highest $CT_{degree}$ were common (focal adhesion) in the distinct datasets (Figure 4 and Supplementary Table S1). Therefore, analysis of $CT_{degree}$ for all the enriched pathways can be expected to provide concrete information about the significance and/or putative targetability of the pathways and its components for a specific disease. In the combined analysis of the DEG list, focal adhesion, PI3K-Akt, MAPK, cAMP, and RAP1 signaling displayed the highest $CT_{degree}$ (Figure 5). For DEGs of the individual dataset, in all the three datasets, focal adhesion was among the top-ranked, highlighting the central role of cell adhesion and the cytoskeleton in coordinating other prostate cancer signaling pathways (Figure 5a–c and Supplementary Table S1). Common to all our analyses, the enriched pathways which were ranked on top did not always correspond to high $CT_{degree}$. In other words, the top-ranked enriched pathway(s) were not always on the top in $CT_{degree}$ ranking. Figure 6 shows the networks of enriched pathways, where the numbers in the nodes indicate the ranking of the enriched pathways (Supplementary Table S1). After analyzing the three datasets separately, we have analyzed the connectivities and the genes (Figure 5b), and the crosstalk between the commonly enriched pathways (Figure 5b,c). Based on these analyses, the putative individual contributors of promoting crosstalk were GSK3B, YWHAE, ATP1A3, GNB2, PRKCA, DLG3, MYC, NCKAP1, MAPT, ACTN2, MAPKAPK2, RELA, and RHEB genes. Likewise, in degree distribution, we observe that a significant number of these DEGs have very high connectivity, which leads to the conclusion that the genes with very high connectivities shared between the pathways are altered most dominantly in prostate cancer, which leads to drastic change in pathway–pathway interactions (crosstalk) and acts as a potential source of this crosstalk, as shown in Figure 5b.

The enriched pathways and $CT_{degree}$ varied to some extent in the three different prostate cancer datasets (Figure 4a). Thus, we further analyzed the common enriched pathways and crosstalk network (pathway–pathway interaction network). Most importantly, among the common enriched pathway list were the well-established pathways known to be altered in different cancer types such as Hippo, regulation of actin cytoskeleton, PI3K-Akt, focal adhesion, and MAPK pathways (Figure 4). In this crosstalk network, we can clearly see the impact of one pathway on another (thickness of the edges stands for $CT_{degree}$) (Figure 5b) with the corresponding $CT_{degree}$ (Figure 5c). Furthermore, Figure 5d presented the detailed view of the genes shared between different pathways and the size of red color circles are based on the connectivity. To understand the crosstalk network at the molecular level, the crosstalk network and the network of DEGs belonging to these pathways were visualized (Figure 5b and Figure 6).

### 3.3. Mathematical Modeling and Simulation, Docking, and Experimental Validation of Pathway Components Obtained from Gene Expression and Network-Based Crosstalk Calculation

**Figure 7:**
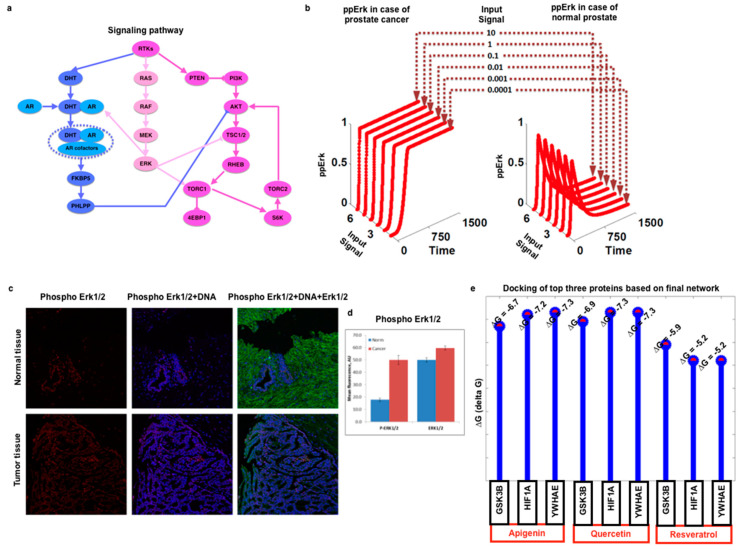
Systems-level application, validation, and integration of high-throughput data: (**a**) A sketch for AKT signaling pathway components in case of prostate cancer, (**b**) kinetics of ppERK calculated by using mass-action kinetics and ODE approach in case of normal and prostate cancer signaling, (**c**) the quantification for immunohistochemistry data, (**d**) ppERK expression in normal human and cancer prostate biopsies, and (**e**) delta G representing the herbal drugs’ interactions with GSK3B, HIF1A, and YWHAE, which appear highly dominant based on high-throughput databased crosstalk result.

After crosstalk calculations, as shown in Figure 5, we have selected a few components which are well-known to play a role in prostate cancer (Figure 7a), prepared a mathematical model by using mass-action kinetics and ordinary differential equations (ODEs), and, by using an evolutionary algorithm (EA), optimized the parameters. Based on mathematical modeling and simulation results, we observe that ppErk (double-phosphorylated or fully activated Erk) are transiently activated in normal prostate signaling, while in the case of prostate cancer, the signaling of ppErk is persistently activated irrespective of the input signal strength (Figure 7b). For further analysis, we have performed immunohistochemistry (IHC) for human prostate biopsies (normal and cancer), and we observe that in normal prostate biopsy samples, there is less expression of ppErk, while in the case of cancer, it is dominantly expressed (Figure 7c,d). We have prepared the model of the well-known genes/proteins for ERK signaling in a precise way to show how we could move from gene expression calculation to pathway enrichment analysis and crosstalk, and finally, we could extend it for the modeling and the simulation of the selective pathway, the selective components, and the verification of selective molecules. As far as the simulation values are concerned, the initial concentration of the signaling molecules was considered as 10 ul, and the kinetic parameters were generated randomly (of very weak strength <0.01). During the evolutionary period, the rate of the reactions was allowed to be any value in the range of 0.01–100. In summary, we could conclude that after gene expression and pathway analysis, we proceeded to the crosstalk analysis, then we moved to the analysis of dynamics of selective target signaling molecules, and finally performed experimental validation. In experimental validation, we selected the MAPK pathway which is dominantly enriched, and in crosstalk analysis, it also appeared to play a pivotal role in prostate cancer. Thus, for the same, we carried out the mathematical modeling and simulation and investigated the expression of the Erk protein in human prostate biopsy samples. Here we clearly observed that the mathematical modeling outcome (Figure 7a,b) and the experimental outcome (Figure 7c,d) for Erk (active and inactive) are closely similar to each other. 

In addition to extending our entire approach, we have integrated our code with AutoDock vina software and also have our own code, which predicts putative binding sites on the protein for the given ligand or drug. Therefore, for more applications in terms of the clinical perspective or personalized therapy, this toolbox can be applied. In this study, three herbal drugs have been selected which are known for their application in herbal cancer therapy (apigenin, quercetin, and resveratrol), targeting the top three proteins which appear to be highly dominant based on our crosstalk analysis (Figure 6); we observe that the delta G values are very close to each other for apigenin and quercetin, which means a higher possibility to have a binding site for the three selected proteins GSK3B, HIF1A, and YWHAE. These values are comparatively lower than that of resveratrol (Figure 7e).

## 4. Discussion

Most of the cellular network architectures have been reported to fall within a scale-free topology [25,26,85]. In this study, the networks were mapped out for the DEGs from the FunCoup network database [51], and the mapped network gives the association of one gene with another as a protein–protein interaction score. After mapping the networks, we consider that one protein is connected with another protein when the protein–protein interaction score was present, i.e., otherwise 0 (no interaction). Thus, the new topology of the network will be with 0 or 1 instead of protein–protein interaction score, which will be a smooth input file for our next step. To find out the design principle of the network and the important network motifs [34,39,86], it is of key importance to understand the network topology, and thus we have analyzed degree distribution and network motifs [34,87]. Importantly, the degree distributions for all the DEGs’ networks, irrespective of the datasets, followed power-law connectivity distribution, which means that a few hubs dominate the overall connectivity of the network (SI1). In network motif analysis, we further found that the same network motif was present in all the three datasets (Supplementary Information Table S1) [88]. Based on our analysis results, we conclude that the top crosstalk pathway, the focal adhesion pathway highly altered in all the three datasets, can dominantly control cell fate decision and functions in prostate cancer by coordinated signals for the changes in cell morphology, as well as growth, migration, and survival dynamics. This was followed by PI3K-AKT as the second top crosstalk pathway in GSE8218 and Grasso datasets. GSK3B, YWHAE, ATP1A3, GNB2, PRKCA, DLG3, MYC, NCKAP1, MAPT, ACTN2, MAPKAPK2, RELA, and RHEB were identified as candidate genes to promote above shown crosstalk.

With the advancement in high-throughput technologies for gene expression, sequencing, and proteomics experiments, the primary goal has been to find the significant gene (differentially expressed or mutated) or protein list and infer the pathways for these lists. For differential gene expression analysis, there are a number of tools available such as DESeq2, DEseq, baySeq2, EBseq, edgeR, etc. [89]. Since we used a microarray dataset for the application of our developed tool for crosstalk analysis and the main goal was to understand the crosstalk, we simply applied the most common approach for differential gene expression. After normalization of raw .cel files, the approach remains similar to DESeq2. The most common approach for inferring the pathways is pathway enrichment analysis by, e.g., DAVID, EnrichNet, and PANTHER analysis tools. From these tools, we get a large number of pathways with significant *p*-values, but this does not clarify how these enriched pathways are associated or crosstalk with each other. By using GECIP, we add yet another step to such pathway-level understanding. The aim of this work is methodology-focused, and off course, there are few steps for which the methodology may resemble many different softwares, such as DEGs’ calculation, motif prediction, docking, and the prediction of enriched pathways, while the main goal was crosstalk (CT-score) prediction and is uniquely presented here. The prediction of crosstalk is completely different from any other work. We agree that elation will be helpful, which we have updated.

Based on our analysis, we consider GECIP particularly useful to translate the high-throughput transcriptomic, genetic, and proteomic data to pathway–pathway-interaction-network-level understanding. As a result, few pathways or representative pathway components with most or least overall crosstalk or connectivity can be selected as targets for more precise studies based on the study questions and goals. Instead of focusing on a large number of pathways, the study focus can thus be narrowed to the selected pathways. Based on the overall analysis results, we conclude that the DEGs and enriched pathways can vary from one dataset to another within the same cancer type, whereas there are also common frequently altered pathways. Notably, all the highly enriched pathways will not have comparable $CT_{degree}$; rather, the pathways ranked low may have high $CT_{degree}$, which means the major affected crosstalk pathways can also be less enriched (Supplementary Table S1). Out of a large list of enriched pathways, there are few highly interconnected pathways which appeared as the central point of transcriptional alterations. Mathematical simulation of selected pathway components and experimental validation also confirms the accuracy of our newly developed approach.

Finally, we conclude that our approach appears unique and novel, which reveals the systems-level understanding and application, validation, and integration of high-throughput data and the possible scope to predict putative drug targets. In summary, we could say that it helps understanding from large biological data integration to phenomics to final drug targets.

## Data Availability

We have used publicly available datasets and the proper references were supplied in the main text and thus could be downloaded from there.

## Supplementary Materials

The following are available online at https://www.mdpi.com/article/10.3390/cells11244121/s1, Figure S1. DEGs network analysis. (a) Prostate cancer DEGs network, (b) degree distribution, and network motif (subgraph). Subgraph (5th) with positive z-score means dominant (highly functional). Table S1: Datasets of all the selected cancer types with accession ID, number of samples, and the platforms for gene expression profiling. References [90,91,92,93,94,95] are cited in Supplementary Materials.
