# Supplementary material for: An Approach for Systems-Level Understanding of Prostate Cancer from High-Throughput Data Integration to Pathway Modeling and Simulation
Source: Cells. 2022 Dec 19;11(24):4121. doi: 10.3390/cells11244121 (PMC9777290; doi:10.3390/cells11244121)
Supplement: Supplementary file 1 [file cells-11-04121-s001.zip › cells-2078052-supplementary.pdf]

Supplementary Information

In this study, we have collected three sets of human gene expression profiling (array) data for mainly three different prostate cancer datasets (Table1) from gene expression omnibus (GEO) database. In addition for cross-validation, we have used the six 1% DEGs sets for prostate cancer from oncomine[90].

Prostate cancer datasets:

(1). GSE1431: It includes 88 organ confined prostate cancer biopsies and in this study normal human biopsies from Yu YP et al.,[91] have been used. We have also used 22 normal prostate biopsies from the same study. The platform used is Human Genome 95Av2. These samples contain prostate carcinoma cells, benign prostatic hypertrophy (BPH) epithelial cells, dilated gland (dilated cystic atrophy) epithelial cells, and stromal cells[92].

(2). GSE8218: This dataset contains 148 samples and 65 are tumors, 71 stoma and without tumor, and 12 are undefined. So, in our study 71 (stromal and no tumor) has been used as control for the 65 tumor samples and the platform is HG-U133A array. In this case148 prostate samples, with different amounts of tumor, stroma, BPH (Benign Prostatic Hyperplasia), and atrophic gland, have been used for this study [93,94].

(3). Grasso: All the samples are HG-U133 plus 2.0 array expression profiling and contains two types of samples 69 without tumor and 85 with tumor [93]. Here, nontumor cases include normal biopsies, normal autopsies, remote stroma as well as pure tumor adjacent stroma.

Table S1. Datasets of all the selected cancer types with accession ID, number of samples, and the platforms for gene expression profiling.

Cancer Types	***Datasets***	***Samples***	***Platform***	***Reference***
Prostate cancer	GSE1431	88	HG-U95Av2	[92]
Prostate cancer	GSE8218	148	HG-U133A	[93,94]
Prostate cancer	oncomine (Grasso)	122	HG-44K	[95]

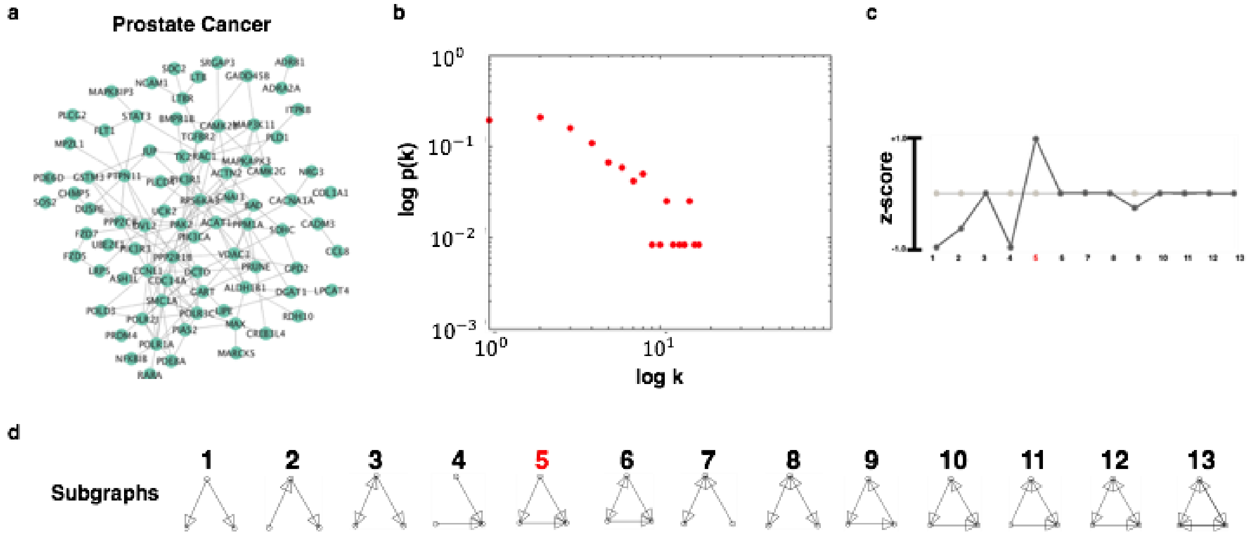

Figure S1. DEGs network analysis. (**a**) Prostate cancer DEGs network, (**b**) degree distribution, and network motif (subgraph). Subgraph (5*th*) with positive z-score means dominant (highly functional).
